# Sex Difference in the Socioeconomic Burden of Osteoporosis among South Koreans

**DOI:** 10.3390/healthcare9101304

**Published:** 2021-09-30

**Authors:** Eun-Whan Lee, Jin Young Nam

**Affiliations:** 1 Gyeonggi Research Institute, Suwon 16207, Korea; ewlee@gri.re.kr; 2 Department of Healthcare Management, Eulji University, Seongnam 13135, Korea

**Keywords:** osteoporosis, economic burden of disease, cost of illness, cost analysis

## Abstract

*Background:* The prevalence of osteoporosis is increasing with the aging of the population and the socioeconomic burden. The purpose of this study was to determine the socioeconomic burden of osteoporosis in Korea. *Methods:* The prevalence of osteoporosis was analyzed using 2017 National Patients Sample and Korea National Health and Nutrition Examination Survey data. Direct costs were divided into healthcare and non-healthcare costs, and indirect costs were calculated by assessing the cost of loss of productivity for labor loss due to disease. *Results:* The prevalence of osteoporosis diagnosis was 1.91% in total, which was 13 times higher in women than in men (3.57% vs. 0.26%). The socioeconomic cost of osteoporosis was 299.1 million USD based on main diagnosis, and the cost was 13 times higher in women than in men (277.6 vs. 21.5 million USD). The total cost based on main and secondary diagnosis was 981.8 million USD. Similarly, the cost was seven times higher in women than in men (862.4 vs. 119.4 million USD). *Conclusions:* Osteoporosis increases the socioeconomic burden of disease, and it is significantly higher in women than in men. The policy support for the implementation of prevention and management programs would be necessary to reduce the burden of osteoporosis.

## 1. Introduction

Osteoporosis is a systemic skeletal disease characterized by decreased bone mass and abnormal microstructure, accompanied by bone fractures [1]. Osteoporosis is an aging-related condition, and thus, its prevalence accordingly increases with age; however, no symptoms are observed until fractures occur. Thus, osteoporosis is also known as “the quiet thief of the bone” [2]. Osteoporosis worsens in the elderly without any symptoms, and the outbreak of symptoms negatively affects both physical and psychological aspects, threatening the patients’ quality of life. The International Osteoporosis Foundation (IOF) reports that 17.8% of Korean patients aged over >50 years die within 1 year of hip fracture [3]. Osteoporosis is highly prevalent in elderly and postmenopausal women [4]. More than 200 million hip fracture cases worldwide are due to osteoporosis [5]. Further, osteoporosis is prevalent in 15% and 30% of men and women aged >50 years, respectively [6]. This indicates that the prevalence and disease burden of osteoporosis are higher in old age groups and in women [2,7,8,9].

Economic loss due to osteoporosis is also increasing worldwide, and fractures due to osteoporosis is also an important factor increasing the burden of the disease [10]. The IOF projects that approximately 300 million people aged >50 years will have osteoporotic fractures by 2040, further increasing the socioeconomic cost [11]. Osteoporosis and fractures not only affect quality of life but also impose a significant economic burden on the health care system [1], including a high medical cost and loss of productivity. A previous study reported an annual related economic loss of 37 billion euros and 19 billion US dollars for the European Nations and the United States, respectively [8,10]. Moreover, osteoporosis and fractures are projected to cause an economic burden of 4.5 million dollars annually by 2025. The total cost of loss, including indirect cost for long-term care and rehabilitation of patients, is expected to increase significantly [10,12,13].

Osteoporosis is becoming a public health concern worldwide owing to its increasing prevalence with the aging of the population and the increasing socioeconomic burden. Therefore, the purpose of this study was to estimate the socioeconomic cost of osteoporosis, including medical expenses and productive loss in Korea, to provide basic evidence for policy- and decision-making on the management of osteoporosis.

## 2. Materials and Methods

### 2.1. Study Design and Data Source

Calculating the exact prevalence is important to estimate the cost of disease. Disease cost is determined based on the validity and accuracy of the estimated prevalence. In this study, a prevalence-based approach was used to calculate the socioeconomic cost of osteoporosis in 2017 [14]. All existing and new patients in the year of analysis were included to determine prevalence [15]. Valuation was conducted using the human capital approach [16]. Productive loss due to loss or reduction of the labor force was expressed as a numerical value based on the income levels by sex and age [17]. Moreover, subjective intervention was excluded, and only objective measures of the socioeconomic burden of osteoporosis were analyzed [15].

Societal perspectives were also included in the analysis [18]. All patient and insurer costs as well as disease-related societal losses were included [14].

Data from the National Patients Sample (NPS) of the Health Insurance Review and Assessment Service (HIRA) in 2017 were used in this study. In Korea, most of the population (98% or higher) is obliged to use the National Health Insurance, and all medical institutions submit insurance claim data to the HIRA. NPS extracts 3% (approximately 1.4 million cases) of the patient data of the HIRA and stores information related to assessment, treatment, and prescription details of the sampled patients for 1 year [19]. The prevalence of osteoporosis diagnosis was calculated by sex and 5-year age brackets based on the main diagnosis using the NPS data. This study adhered to the tenets of the Declaration of Helsinki and was reviewed and approved by the ethics board of Eulji University (EU21-042).

### 2.2. Case Definition

In the National Health Insurance big data, osteoporosis was defined as diagnostic codes M80, M81, and M82 following the International Statistical Classification of Diseases and Related Health Problems. As osteoporosis does not have specific symptoms, it was defined as a case in which the main diagnosis and main or secondary diagnosis was osteoporosis in the health insurance data.

### 2.3. Cost Components

The components of direct and indirect costs were used to calculate the socioeconomic cost of osteoporosis, as described in previous studies [20,21]. The components of socioeconomic costs are shown in Table 1.

### 2.4. Estimation Method

Socioeconomic costs were measured using direct and indirect costs. Direct costs were calculated by analyzing data from the NPS and included direct healthcare costs (DHC) and direct non-healthcare costs (DNHC). NPS data included health insurance covered costs. Thus, health insurance noncovered costs were applied at 18.0% for inpatient cost and 24.5% for outpatient cost, as suggested in the survey on the benefit coverage rate of the National Health Insurance [22]. The medical care cost was calculated as:

Medical care cost = [inpatient cost × 0.18/(1 − 0.18)] + [outpatient cost × 0.245/(1 − 0.245)]

DHC was calculated as:

(1) $$\mathrm{Direct}\;\mathrm{healthcare}\;\mathrm{cost}=\underset{s}{\sum }\underset{y}{\sum }\left[E_{isy}\left(1+\alpha \right)+E_{osy}\left(1+\beta \right)\right]$$

where *i* indicates inpatient; *s*, sex; *o*, outpatient; *y*, age; *α*, non-health insurance benefit cost rate for inpatient; *β*, non-health insurance benefit cost rate for outpatient; and *E*, medical expenses.

DNHC included transportation expenses, nursing expenses for hospitalization, and opportunity costs of the guardians, assuming the patients are accompanied by a guardian for their outpatient visits [23]. The transportation cost was 9.7 US dollars for outpatients and 12.0 US dollars for inpatients and was determined using the average transportation cost presented in the 2005 Korea National Health and Nutrition Examination Survey by the Korea Disease Control and Prevention Agency and the increased rate of the transportation cost presented in the 2017 Consumer Price Survey by Statistics Korea [24]. Transportation cost was calculated using the following formula:

Transportation cost = number of hospital visits × average cost of one − a way trip × price index in 2017 × 2 (round trip)

For nursing cost and opportunity cost in instances when patients were accompanied by a guardian, we used the cost per person reported by the Korea Caregiver Association in 2017. Hospitalization was calculated based on the length of hospital stay. For outpatients, the time of outpatient visit was calculated as 1/3 of the daily working hours. The caregiver cost was calculated using the following formula:

Caregiver cost = caregiver cost per day × (length of hospital stay + 1/3 number of outpatient visits)

Meanwhile, DNHC of osteoporosis was calculated as follows:

(2) $$\mathrm{Direct}\;\mathrm{non}-\mathrm{healthcare}\;\mathrm{cost}=\underset{s}{\sum }\underset{y}{\sum }\left[\left(N_{isy}+N_{osy}\right)\times C_{t}\times 2\right]+\underset{s}{\sum }\underset{y}{\sum }[\left(L_{sy}\times C_{c}\right)+\frac{1}{3}\left(N_{osy}\times C_{c})\right]$$

where *i* indicates inpatient; *s*, sex; *o*, outpatient; *y*, age; C_c_, caregiver cost; *N*, number/count of cases; C_t_, transportation cost; and *L*, length of hospital stay.

Direct cost indicated the loss of labor time (reduction of working hours) due to illness. The data of those who were within the legal working age of 15–69 years in Korea were included in the calculation. The average wage and employment by sex and age of the economic population in Korea, provided by the Korean Ministry of Employment and Labor, were also applied to calculate the direct costs. For inpatients, the average daily wage by age group was applied to the length of hospital stay. For outpatients, the average wage was applied to the length of hospital stay, and lost time per day was calculated as 1/3 of the working hours. The cost of productivity loss due to osteoporosis was calculated using the following formula:

(3) $$\mathrm{Cos}t\;\mathrm{of}\;\mathrm{productivity}\;\mathrm{loss}=\underset{s}{\sum }\underset{y}{\sum }\left[\left(N_{isy}+\frac{1}{3}N_{osy}\right)\times W_{sy}\times E_{sy}\right]$$

where *i* indicates inpatient; *s*, sex; *o*, outpatient; *y*, age; *W*, average wage; *N*, number/count of cases; and *E*, employment rate.

All statistical analyses were performed using SAS 9.4 (SAS Institute, Inc., Cary, NC, USA).

## 3. Results

### 3.1. Prevalence and Economic Burden of Osteoporosis by Sex and Age

**Figure 1:**
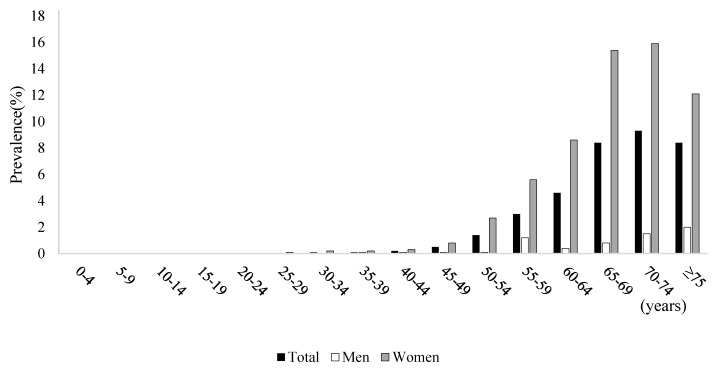
Prevalence based on principal diagnosis and economic burden of osteoporosis by sex and age.

**Figure 2:**
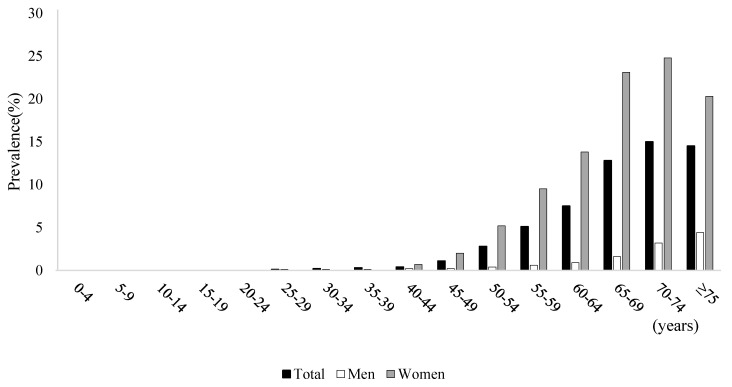
Prevalence based on principal or second diagnosis and economic burden of osteoporosis by sex and age.

Based on NPS data, the overall prevalence of osteoporosis diagnosis in 2017 was 1.91% (*n* = 980,322 cases). The prevalence of osteoporosis in women was 3.57% (*n* = 914,490 cases), and this was approximately 13 times higher than that in men (0.26%, *n* = 65,832) (Figure 1 and Supplementary Table S1). The prevalence of osteoporosis diagnosis in people aged 50 years or more was 3.44% in women and 0.23% in men (Supplementary Table S1). Meanwhile, based on main/secondary diagnosis, the overall prevalence of osteoporosis diagnosis was 3.21% (*n* = 1,649,782 cases). The prevalence of osteoporosis diagnosis in women was 5.90% (1,509,751 cases), and this was approximately 10 times higher than that in men (0.54%, *n* = 140,031 cases) (Figure 2 and Supplementary Table S2). The prevalence of osteoporosis diagnosis in people aged 50 years or more was 5.58% in women and 0.49% in men (Supplementary Table S2).

### 3.2. Economic Burden of Osteoporosis by Cost Components

The total cost of osteoporosis is shown in Table 2. The total cost, including DHC, DNHC, and indirect costs based on the main diagnosis was 299.1 million USD. The total cost was approximately 13 times higher in women than in men at 21.5 million USD versus 277.6 million USD. Comparison by sex and age showed that the total cost of osteoporosis was the highest in both men and women aged ≥75 years at 86.6 million USD and 8.0 million USD, respectively.

Meanwhile, the total cost including DHC, DNHC, and indirect costs based on main/secondary diagnosis was approximately 981.8 million USD. The total cost was 862.4 million USD in women, and this was approximately seven times higher than that in men at 119.4 million USD. Comparison by sex and age showed that the total cost of osteoporosis was the highest in both men and women aged ≥ 75 years at 133.9 million USD and 45.7 million USD, respectively (Table 2).

## 4. Discussion

Osteoporosis is becoming a public health concern worldwide. In this study, the socioeconomic cost of osteoporosis based on the main diagnosis was 13 times higher in women than in men. In addition, the socioeconomic cost based on the main and secondary diagnosis was seven times higher in women than in men. This was equivalent to 0.018–0.061% of Korea’s gross domestic product in 2017.

The socioeconomic cost of osteoporosis is similar to that of diabetes mellitus (approximately 1165.2 million USD) and breast cancer (974.9 million USD) and is approximately three times higher than that of cervical cancer (approximately 336.5 million USD) and allergic rhinitis (approximately 285.5 million USD) [15,25,26]. A previous study using NHANES data showed that osteoporosis was prevalent in 12% of the elderly aged over 80 years, and the prevalence was higher in women than in men (77.1% vs. 46.3%) [27]. In our study, the prevalence of osteoporosis in the elderly aged over 65 years was 26.14%, and this rate was approximately 10 times higher in women than in men (43.39% vs. 4.31%). This higher prevalence in women is primarily because 80% of primary osteoporosis is caused by estrogen deficiency [4,28]. In addition, type 2 primary osteoporosis is mainly caused by aging and is observed mostly after age 70 [2]. Aging leads to a decreased production of 1,25(OH)_2_ vitamin D and calcium intake deficiency, which ultimately causes osteoporosis [29,30].

Our results also showed that the total cost of osteoporosis based on main diagnosis and main/secondary diagnosis was higher in the elderly aged 65–75 years. A US study in 2005 demonstrated that the direct medical costs of osteoporosis, including fractures, were $17 billion, with a total cost of over $19 billion. It was predicted that the total annual cost of osteoporosis in the US would reach 25.3 billion in 2025. In particular, the study showed that more than 87% of the elderly aged 65–74 years are at a high risk of fractures [10]. Burge et al. (2007) estimated the annual total cost of fractures of the hip, spine, wrist, pelvis, and other bones and reported that 57%, 13%, and 30% of the fractures led to inpatient treatment, outpatient treatment, and long-term treatment, respectively [10]. Moreover, hip fractures accounted for 72% of the total cost, while accidental fractures accounted for only 14% [13]. The bone mineral density of the femur is reduced by approximately 1% annually after age 50, and the risk of hip fracture increases with the decrease in bone mineral density [31]. Particularly, elderly women have a higher risk of osteoporosis than elderly men. Thus, regular education on fall prevention is necessary.

This study has some limitations. First, NPS data, which represent samples of a real patient population, were used. Thus, the results have limited accuracy because it was an estimated rather than an actual value. However, there were no significant differences between the direct medical costs calculated in this study and the annual medical expenses reported by the NHI. Second, there were noncovered costs included in the direct medical costs. Direct medical costs include the insurer’s payment, copayment (out-of-pocket patient expense), and uninsured expenses. In this study, the noncovered cost was not an actual value but an estimated value obtained from a survey of medical expenses of the patients. Given that there are currently no data regarding the noncovered costs in South Korea, there are uncertainties in the noncovered costs. Last, the total costs of osteoporosis may be related to difference between fracture and non-fracture. However, this study did not consider whether fractures existed or not, or what type of fractures existed. These factors directly affect the cost differences, but this study did not include specific information of fracture/non-fracture patients with osteoporosis. Further research should be conducted on the difference between more specific types of fracture with osteoporosis.

Despite these limitations, this study also has several strengths. First, to our best knowledge, this study is the first to calculate the economic burden of osteoporosis in the Korean population using data from a sample population and the National Health and Nutrition Examination Survey, which has a representative record of the Korean population. Second, this study adopted a social point of view and calculated socioeconomic costs including the loss of labor force and national productivity loss due to osteoporosis. Previous studies assessed the cost only from the viewpoint of insurers or patients. Moreover, previous studies calculated only the direct costs. However, in this study, we calculated the cost of osteoporosis from a socioeconomic perspective. Our data on socioeconomic loss can be used as major indicator to set health policy priorities in the country. Finally, the burden of disease was presented in monetary value, which would help the general public and policy makers to easily understand the socioeconomic cost of osteoporosis.

## 5. Conclusions

Osteoporosis increases the socioeconomic burden of disease, and it is significantly higher in women than in men in South Korea. The results of this study provide the basic evidence for policy- and decision-making on the management of osteoporosis. Therefore, the policy support for the implementation of prevention and management programs would be necessary to reduce the burden of osteoporosis.

## Figures and Tables

**Table 1:** Components and definitions of socioeconomic costs of osteoporosis.

Category of Costs		Components of Costs		Definition
Direct costs	Direct healthcare costs	Medical institutions	Covered cost	Cost confirmed by HIRA and paid by insurers
			Out-of-pocket payment	Cost confirmed by HIRA and paid by patients
			Noncovered cost	Cost of uncovered services paid by patients
	Direct non-healthcare costs	Transportation		Cost of transportation for visiting hospital
		Caregiving		Cost of hiring caregivers and opportunity costs for family members for outpatient visits
Indirect costs	Losses of productivity	Hospitalized patients		Cost of reducing working hours for medical care
		Outpatients		
		Premature death		Cost of losing labor due to premature death

**Table 2:** Socioeconomic costs of osteoporosis by cost components in 2017 (USD).

	Principal Diagnosis			Principal/Second Diagnosis		
	Total	Male	Female	Total	Male	Female
DHC						
Inpatient care	19.3	2.3	17.0	278.6	40.5	238.1
Outpatient care	139.0	7.2	131.7	327.9	33.4	294.5
NHC						
Transportation	32.5	2.0	30.5	64.9	4.9	60.0
Caregiving	82.7	5.9	76.8	244.9	26.7	218.2
Indirect cost						
PL	25.6	3.9	21.7	65.5	13.9	51.6
Total	299.1	21.5	277.6	981.8	119.4	862.4

Abbreviation: DHC, direct healthcare costs; NHC, direct non-healthcare costs; PL, losses of productivity. The Won–USD exchange rate in 2017 was $1 = 1070.5 Won.

## Data Availability

Restrictions apply to the availability of these data. Data were obtained from https://opendata.hira.or.kr/home.do (accessed on 26 August 2021) and are available from the authors with the permission of https://opendata.hira.or.kr/home.do (accessed on 26 August 2021).

## Supplementary Materials

The following are available online at https://www.mdpi.com/article/10.3390/healthcare9101304/s1, Table S1: Prevalence based on principal diagnosis and economic burden of osteoporosis by sex and age, Table S2: Prevalence based on principal or second diagnosis and economic burden of osteoporosis by sex and age.
