# Supplementary material for: Sex Difference in the Socioeconomic Burden of Osteoporosis among South Koreans
Source: Healthcare (Basel). 2021 Sep 30;9(10):1304. doi: 10.3390/healthcare9101304 (PMC8544502; doi:10.3390/healthcare9101304)
Supplement: Supplementary file 1 [file healthcare-09-01304-s001.zip › healthcare-1375845-supplementary.pdf]

Table S1. Prevalence based on principal diagnosis and economic burden of osteoporosis by sex and age.

Age Group (Years)	Prevalence, *n* (%)		
	Total	Men	Women
0–4	33 (0.00)	33 (0.00)	0 (0.00)
5–9	0 (0.00)	0 (0.00)	0 (0.00)
10–14	67 (0.00)	0 (0.00)	67 (0.01)
15–19	300 (0.01)	200 (0.01)	100 (0.01)
20–24	500 (0.01)	133 (0.01)	367 (0.02)
25–29	1467 (0.04)	167 (0.01)	1300 (0.08)
30–34	3367 (0.10)	533 (0.03)	2833 (0.17)
35–39	4467 (0.11)	1067 (0.05)	3400 (0.17)
40–44	7100 (0.17)	1533 (0.07)	5567 (0.28)
45–49	21,167 (0.47)	2533 (0.11)	18,633 (0.84)
50–54	59,799 (1.44)	3600 (0.17)	56,199 (2.73)
55–59	125,932 (2.98)	6233 (0.30)	119,699 (5.64)
60–64	145,966 (4.60)	6900 (0.44)	139,066 (8.61)
65–69	191,795 (8.39)	8800 (0.80)	182,996 (15.41)
70–74	163,966 (9.33)	12,167 (1.52)	151,799 (15.87)
≥75	254,398 (8.42)	21,933 (1.99)	232,465 (12.11)
Total	980,322 (1.91)	65,832 (0.26)	914,490 (3.57)

Table S2. Prevalence based on principal or second diagnosis and economic burden of osteoporosis by sex and age.

Age Group (Years)	Prevalence, *n* (%)		
	Total	Men	Women
0–4	100 (0.00)	67 (0.01)	33 (0.0)
5–9	267 (0.01)	100 (0.01)	167 (0.0)
10–14	400 (0.02)	267 (0.02)	133 (0.0)
15–19	967 (0.03)	533 (0.03)	433 (0.0)
20–24	1533 (0.04)	567 (0.03)	967 (0.1)
25–29	3567 (0.11)	900 (0.05)	2667 (0.2)
30–34	7433 (0.21)	1133 (0.06)	6300 (0.4)
35–39	10,300 (0.25)	2467 (0.12)	7833 (0.4)
40–44	18,000 (0.44)	3200 (0.15)	14,800 (0.7)
45–49	48,266 (1.07)	4533 (0.20)	43,733 (2.0)
50–54	114,999 (2.76)	7667 (0.36)	107,332 (5.2)
55–59	214,898 (5.09)	12,500 (0.59)	202,398 (9.5)
60–64	236,665 (7.46)	14,400 (0.93)	222,265 (13.8)
65–69	292,326 (12.78)	17,733 (1.61)	274,594 (23.1)
70–74	262,899 (14.96)	25,767 (3.22)	237,132 (24.8)
≥75	437,163 (14.46)	48,199 (4.37)	388,964 (20.3)
Total	1,649,782 (3.21)	140,031 (0.54)	1,509,751 (5.90)
